# Burden and Predictors of Depression in Populations With Coronary Heart Disease

**DOI:** 10.7759/cureus.62068

**Published:** 2024-06-10

**Authors:** Emmanuel O Ilori, Chinaza Erechukwu, Vivien O Obitulata-Ugwu, Zimakor D Ewuzie, Okelue E Okobi, Oluwatosin B Iyun

**Affiliations:** 1 Psychiatry and Behavioral Sciences, Garnet Health Medical Center, Middletown, USA; 2 Public Health, University of East London, London, GBR; 3 College of Medicine, University of Nigeria, Enugu, NGA; 4 Adult Psychiatry, Cygnet Hospital Harrogate, Harrogate, GBR; 5 Family Medicine, Larkin Community Hospital Palm Springs Campus, Miami, USA; 6 Family Medicine, Medficient Health Systems, Laurel, USA; 7 Family Medicine, Lakeside Medical Center, Belle Glade, USA; 8 School of Public Health and Family Medicine, University of Cape Town, Cape Town, ZAF

**Keywords:** cigarette, insurance, age, coronary heart disease, depression

## Abstract

Introduction: Depression significantly impacts the quality of life and medical care in patients with coronary heart disease (CHD). This study assesses the burden of depression in adults aged 40 years and above with CHD and evaluates predictors of depression in this population. It has been reported that approximately 17-44% of persons with CHD have a major depression diagnosis and that nearly 27% of individuals undergoing coronary artery bypass graft operation suffer depression following the procedure.

Methods: Data from the 2022 National Health Interview Survey was used. The sample was made up of adults 40 years and above with CHD. A chi-square analysis was used to identify differences between those who were depressed and those who were not. Logistic and ordinal regression analyses were used to identify predictors of depression and severe depression, respectively.

Results: The proportion of adults 40 years and above with CHD who reported having depression was 863/1700 (50.5%). Among those who were ≥65, the proportion of those who reported depression and those who did not were similar (49.3% vs. 50.7%). Most women reported having depression (57.4% vs. 42.6%), while fewer men reported having depression (46.3% vs. 53.7%). The positive predictors of depression include being insured (odds ratio (OR) 1.26 (1.05-1.53), p = 0.016), college degree (OR 1.09 (1.01-1.18), p = 0.040), diabetes mellitus (OR 1.28 (1.15-1.42), p < 0.001), and hypertension (OR 1.34 (1.24-1.44), p < 0.001). The negative predictors of being depressed were age ≥65 (OR 0.74 (0.69-0.80), p < 0.001), male sex (OR 0.54 (0.50-0.58), p < 0.001), and ratio of family income (RFI) ≥1 (OR 0.68 (0.61-0.77), p < 0.001). The positive predictors of severe depression include diabetes mellitus (OR 1.38 (1.06-1.81), p = 0.019) and current cigarette use (OR 2.10 (1.44-3.07), p < 0.001).

Conclusion: A significant proportion of adults 40 years and above with CHD have depression, and socioeconomic and cardiovascular risk factors are associated with a high likelihood of depression. Cardiovascular risk factors alone predict the likelihood of severe depression. Interventions to address depression in CHD should target specifically these high-risk individuals.

## Introduction

Depression and coronary heart disease (CHD) have a bidirectional relationship; depression is a risk factor for CHD, and CHD predisposes individuals to depression [1]. The mechanism by which depression increases the risk of CHD has been shown to be multifactorial. Behavioral characteristics such as smoking and sedentary lifestyle, which are common in populations with depression and are also traditional cardiovascular risk factors, have been implicated. In addition, functional impairment among the depressed, which leads to low adherence to medical therapy and reduced willingness to seek medical care, has been mentioned as contributing factors [2-4].

In addition to behavioral and functional factors, physiological contributors have also been identified. They include an increase in inflammatory markers, endothelial dysfunction, increased platelet activity and aggregation, and neurohormonal and autonomic nervous system dysfunctions [5]. Thus, depression leads to an interplay of several factors, which culminates in a two- to 2.5-fold increase in the risk of mortality in patients with CHD [3]. It is therefore important to identify patients with CHD who have depression so that appropriate interventions addressing it can be instituted.

Studies that have evaluated the predictors of depression in CHD have shown that gender, illness perception, emotional social support, and perception of cardiac ischemic symptoms are some of the predictors of depression in populations with CHD [6,7]. These studies were however conducted using small samples and a nationally representative study of the United States (US) population is lacking. In this study, we sought to evaluate the burden of depression in adults 40 years and above with CHD and assess the predictors of depression in general and severe depression in a nationally representative sample of the US.

## Materials and methods

Data

Data from the 2022 National Health Interview Survey (NHIS) were used for this study. The NHIS is conducted continuously throughout the year by the National Center for Health Statistics (NCHS). Its main objective is the monitoring of the health of the population through the collection and analysis of data on a broad range of health topics. Interviews are typically conducted in respondents’ homes, but follow-ups to complete interviews may be conducted over the telephone. Further information about these NHIS data is available online [8].

Study design

This study employs a cross-sectional design to assess the burden and predictors of depression in adults aged 40 years and above with CHD. The cross-sectional research design has been chosen for this study for several reasons, including the observation that the research design is appropriate for population-based studies, and the assessment of the prevalence of depression in populations with coronary heart disease. In addition, the research design was selected as it enables researchers to carry out studies faster and in an inexpensive way.

Study population

A total of 27,651 respondents were asked if they had ever been told they had CHD. Of these, 76 (<1.0%) did not respond to the question and were excluded. Among those who responded, 7,945 were below age 40 and were also excluded since CHD is rare in this age group [9], leaving a population of 19,630/27,651. Among them, 1,700/19,630, representing 12,183,650 US adults, reported having CHD.

Sociodemographic variables

Sociodemographic variables were categorized as follows: age (40-64 and ≥65 years), ethnicity (non-Hispanic (NH) White, Hispanic, NH black, and NH others), gender (female and male), level of education (graduate from college and did not graduate from college), family income to poverty ratio (FIPR) (<1 and ≥1), and insurance status (uninsured and insured).

Comorbidities

Respondents were asked about their medical comorbidities, categorized as diabetes mellitus (yes/no), hypertension (yes/no), and smoking status (never, current, former).

Depression

Respondents with depression were identified from the variable “level of how depressed.” Those who responded with “a little, a lot, or somewhere in between a little and a lot” were categorized as depressed. Those who responded otherwise were categorized as not depressed. They also had a patient health questionnaire (PHQ) scale categorization as “none/minimal, mild, moderate, severe, and not ascertained.” For this study, this was re-categorized as none/minimal, mild-moderate, and severe.

Statistical analyses

Sampling weights were applied during the analysis to account for the survey’s complex study design. A Pearson’s chi-square was used to determine group differences by depression status among respondents with CHD with counts and weighted proportions reported. A logistic regression analysis was conducted to establish the predictors of depression in the study population, followed by an ordinal regression analysis to identify the relationship between respondent characteristics and the likelihood of having severe depression. Stata/SE 17.0 (Stata Corp LLC, College Station, TX, USA) was used for statistical analysis, and p < 0.05 was set for a significance level in a two-tailed test.

## Results

The characteristics of the study population are shown in Table 1. Half (50.5%) of adults aged 40 years and above with CHD reported having depression. Among those who were 40-64 years, a larger proportion reported being depressed (53.2% vs. 46.9%), while among those who were ≥65, the proportion of those who reported depression and those who did not were similar (49.3% vs. 50.7%). Most women reported having depression (57.4% vs. 42.6%), while fewer men reported having depression (46.3% vs. 53.7%). More Hispanics reported depression (54.4% vs. 45.6%), while the proportion of NH Black/AA who did not have depression was higher than those who did (47.8% vs. 52.2%). Among those who belonged to the ratio of family income (RFI) group of <1, a higher proportion reported depression (60.7% vs. 39.3%). For the uninsured, a small proportion reported depression (37.6% vs. 62.4%), while a larger proportion of those who currently use cigarettes reported depression (60.3% vs. 39.7%).

**Table 1:** Background characteristics of the study population n (%): sample size (percentage), NH White: non-Hispanic White, NH black/AA: non-Hispanic Black/African American, RFI: ratio of family income poverty ratio, CI: confidence interval

Variables		Total (n = 1700) (100%)	No depression (n = 837) (49.5%)	Depression (n = 863) (50.5%)	p-value
Age in years	40–64	393 (100)	169 (46.9)	224 (53.2)	0.275
	≥65	1307	668 (50.7)	639 (49.3)	
Sex	Female	705 (100)	298 (42.6)	407 (57.4)	<0.001
	Male	995 (100)	539 (53.7)	456 (46.3)	
Ethnicity	NH White	1317 (100)	652 (49.8)	665 (50.2)	0.785
	Hispanic	123 (100)	53 (45.6)	70 (54.4)	
	NH black/AA	169 (100)	85 (52.2)	84 (47.9)	
	Others	91 (100)	47 (48.3)	44 (51.7)	
RFI	<1	234 (100)	94 (39.3)	140 (60.7)	0.002
	≥1	1466 (100)	743 (51.1)	723 (48.9)	
Insurance	Uninsured	26 (100)	14 (62.4)	12 (37.6)	0.236
	Insured	1674 (100)	823 (49.3)	851 (50.7)	
Level of education	No college degree	1198 (100)	572 (48.3)	626 (51.7)	0.099
	College degree	493 (100)	261 (53.3)	232 (46.7)	
Diabetes mellitus	No diabetes mellitus	1190 (100)	604 (51.5)	586 (48.5)	0.045
	Diabetes mellitus	505 (100)	231 (45.3)	274 (54.7)	
Hypertension	No hypertension	359 (100)	196 (54.3)	163 (45.7)	0.076
	Hypertension	1341 (100)	641 (48.3)	700 (51.7)	
Smoking status	Never	757 (100)	400 (51.6)	357 (48.4)	0.037
	Current	210 (100)	79 (39.7)	131 (60.3)	
	Former	687 (100)	331(49.5)	356 (50.5)	

Table 2 shows the predictors of depression in adults 40 years and above with CHD. The positive predictors of depression in adults 40 years and above with CHD include being insured (OR 1.26 (1.05-1.53), p = 0.016), having a college degree (OR 1.09 (1.01-1.18), p = 0.040), diabetes mellitus (OR 1.28 (1.15-1.42), p < 0.001), hypertension (OR 1.34 (1.24-1.44), p < 0.001), and use of cigarettes. The negative predictors of being depressed were age ≥65 (OR 0.74 (0.69-0.80), p < 0.001), male sex (OR 0.54 (0.50-0.58), p < 0.001), minority race, and RFI ≥1 (OR 0.68 (0.61-0.77), p < 0.001).

**Table 2:** Predictors of depression in adults 40 years and above with CHD NH White: non-Hispanic White, NH black/AA: non-Hispanic Black/African American, RFI: ratio of family income poverty ratio, CI: confidence interval, CHD: coronary heart disease

Variables		Odds ratio (95% CI)	p-value
Age in years	40–64	Reference	<0.001
	≥65	0.74 (0.69–0.80)	
Sex	Female	Reference	<0.001
	Male	0.54 (0.50–0.58)	
Ethnicity	NH White	Reference	<0.001
	Hispanic	0.68 (0.61–0.76)	
	NH black/AA	0.69 (0.61–0.78)	
	Others	0.67 (0.58–0.78)	
RFI	<1	Reference	<0.001
	≥1	0.68 (0.61–0.77)	
Insurance	Uninsured	Reference	<0.001
	Insured	1.26 (1.05–1.53)	
Level of education	No college degree	Reference	<0.001
	College degree	1.09 (1.01–1.18)	
Diabetes mellitus	No diabetes mellitus	Reference	<0.001
	Diabetes mellitus	1.28 (1.15–1.42)	
Hypertension	No hypertension	Reference	<0.001
	Hypertension	1.34 (1.24–1.44)	
Smoking status	Never	Reference	<0.001
	Current	1.81 (1.61–2.04)	
	Former	1.41 (1.30–1.53)	

Table 3 shows the predictors of having severe depression among adults 40 years and above with CHD. The positive predictors of severe depression include diabetes mellitus (OR 1.38 (1.06-1.81), p = 0.019) and current cigarette use (OR 2.10 (1.44-3.07), p < 0.001). The negative predictors of severe depression include age ≥65 (OR 0.62 (0.46-0.83), p = 0.001), male sex (OR 0.50 (0.39-0.65), p = 0.001), and having a college degree (OR 0.71 (0.54-0.94), p = 0.016).

**Table 3:** Predictors of severe depression in adults 40 years and older with CHD NH White: non-Hispanic White, NH black/AA: non-Hispanic Black/African American, RFI: ratio of family income poverty ratio, CI: confidence interval, CHD: coronary heart disease

Variables		Odds ratio (95% CI)	p-value
Age in years	40–64	Reference	-
	≥65	0.62 (0.46–0.83)	0.001
Sex	Female	Reference	-
	Male	0.50 (0.39–0.65)	0.001
Ethnicity	NH White	Reference	-
	Hispanic	1.44 (0.91–2.27)	0.120
	NH Black/AA	0.85 (0.57–1.25)	0.407
	Others	1.08 (0.61–1.93)	0.794
RFI	<1	Reference	-
	≥1	0.78 (0.55–1.10)	0.153
Insurance status	Uninsured	Reference	-
	Insured	2.20 (0.59–8.25)	0.242
Level of education	No college degree	Reference	-
	College degree	0.71 (0.54–0.94)	0.016
Diabetes mellitus	No diabetes mellitus	Reference	-
	Diabetes mellitus	1.38 (1.06–1.81)	0.019
Hypertension	No hypertension	Reference	-
	Hypertension	1.38 (0.99–1.92)	0.054
Smoking status	Never	Reference	-
	Current	2.10 (1.44–3.07)	<0.001
	Former	1.24 (0.94–1.65)	0.128

## Discussion

In this study, we sought to evaluate the burden of depression in adults 40 years and above with CHD and assess the predictors of depression and its severe form. Our study found that half of the US population aged 40 years and above with CHD has depression. Socioeconomic and traditional cardiovascular risk factors are positive predictors of depression, while advanced age and male sex were negatively associated with having depression. Furthermore, among those who reported depression, some cardiovascular risk factors, namely, diabetes mellitus and current cigarette use, were associated with severe depression, while advanced age and male sex were negatively associated with having severe depression.

One in two US adults 40 years and above with CHD reported having depression. A lower prevalence of depression among patients with CHD was reported in a systematic review of four studies conducted in China [10] and in a cross-sectional descriptive study of adults with CHD in Iran [11]. A similar prevalence of depression in CHD populations was however reported in an Australian study [12]. In the former studies, the sample size was small and hence may explain why the prevalence was lower as compared to what was observed in this study. However, when the findings from our study are compared to the second study, the higher prevalence of depression in the US may be related to sociodemographic correlates, such as role transitions and functional societal roles, that differ between the US and Iran [13]. When compared to the study from Australia, the similar prevalence of depression may be due to similar sociodemographic correlates, like the distribution of education and income between the US and Australia. Nevertheless, having half of the US population with CHD also have depression is a cause for concern, and it highlights this problem as a public health emergency.

A higher socioeconomic status characterized by having a college degree and being insured was associated with reports of depression. This is in contrast to the inverse relationship between socioeconomic status and depression reported in other studies [14,15]. In addition, we found that traditional cardiovascular risk factors were associated with being more likely to report having depression in general and even more so in its severe form. In the general population, diabetes mellitus and hypertension have been shown to increase the risk of depression [16-18], and it appears a similar relationship exists in CHD populations as shown in this study. This observation in CHD populations has previously been reported by Murphy et al., but in their study, it was considered to be less significant as compared to the effect of socioeconomic status [14]. Thus, in patients with CHD, paying attention to socioeconomic and comorbid characteristics will not only help identify those who have depression but could also help guide the provision and application of interventions to manage this condition given the increased risk of adverse cardiovascular outcomes in patients with depression and CHD.

However, in this study, not all markers of a high socioeconomic status were associated with being more likely to report depression. A better ratio to family income poverty ratio was associated with being less likely to report depression. This builds upon previous cohort and cross-sectional studies that have shown an inverse relationship between income and depression [19,20]. Thus, illustrating that different elements of socioeconomic status may impact the risk of depression differently in populations with CHD. In addition, the male sex was not only associated with a less likelihood of reporting depression but was a negative predictor of having severe depression. This supports reports from previous studies of the association between the female sex and severe depression [21,22]. An explanation may be a result of the sex differences in biology and gender roles, which increases the likelihood of females experiencing a more severe form of depression [23]. Considering the relationship of income and gender with depression, populations with CHD in these categories can be targeted for detailed screening and interventions to reduce their risk.

This study not only has several strengths, including the use of a nationally representative sample, but also several limitations. We made use of a nationally representative sample of the US using sampling weights in our analysis; therefore, estimates can be extrapolated to national levels. We considered important socioeconomic and comorbid variables in assessing the predictors of depression in populations with CHD. However, some other variables such as illness perception and CHD symptoms and their relationship with the risk of depression were not assessed. Although we made use of a survey question that assessed how depressed respondents feel it is difficult to draw conclusions that these respondents had a clinical diagnosis of depression, and as such, we must be careful as we apply the findings of this study. This is further compounded by the nature of the data. Given that the survey data were used in the analysis, the possibility of recall bias exists; thus, the diagnosis of severe depression may not be accurate.

## Conclusions

One in two US adults aged 40 years and above with CHD have depression, highlighting a significant public health concern due to the relationship between depression and adverse cardiovascular outcomes. This finding highlights the need for effective interventions to minimize depression in this population. In addition, this highlights the need for comprehensive clinical and experimental studies to determine the best pharmacological and non-pharmacological interventions, as well as to enable the development of effective treatment guidelines for the management of depression in CHD. Moreover, in these groups of patients, paying attention to traditional cardiovascular risk factors can help identify populations with depression for targeted interventions. Furthermore, in identifying populations with CHD who may have depression, its relationship with different socioeconomic factors should be taken into consideration to guide interventions and strategies to reduce the burden of depression in this population. Although an important association between gender and severe depression, which is consistent with existing literature, was found, we must be cautious as we apply these findings due to the limitations of the study design. Further studies are needed to evaluate the identified relationships as more efforts are made by all stakeholders to address depression in populations with CHD.
